# The effects of single versus multiple training sessions on the motor learning of two Krav Maga strike techniques, in women

**DOI:** 10.7717/peerj.8525

**Published:** 2020-02-13

**Authors:** Vincenzo E. Di Bacco, Mehran Taherzadeh, Olivier Birot, William H. Gage

**Affiliations:** School of Kinesiology and Health Science, York University, Toronto, ON, Canada

**Keywords:** Krav Maga, Self-defense, Motor learning, Biomechanics, Women

## Abstract

**Background:**

Experts of the Krav Maga (KM) self-defense system propose that KM techniques are based on simple body movements which are suggested to be learned rapidly and retained. This study investigated the acquisition, retention, and further improvement with additional training of two KM strike techniques among novice female practitioners: straight punch and defensive kick.

**Methods:**

Sixteen healthy females (age: 23 ± 3.7 years) without any previous martial arts/self-defense experience volunteered to participate. All participants received an initial 30-min instruction session (AQ), taught by a certified KM instructor, where each technique was deconstructed into three checkpoints (defined as a component of the entire movement) for learning. Participants were divided into two groups, one of which received additional training. Several kinematic and kinetic measures were recorded at four timepoints: immediately before AQ, immediately after AQ, 5 days after AQ, and 12 days after AQ.

**Results:**

Results suggest that both techniques were learned rapidly, as checkpoint performance was significantly improved after AQ. Kick velocity and impact force also increased significantly after AQ; however, these measures did not change after AQ for the punch technique. Additional training did not improve either punch or kick performance beyond that learned during AQ.

**Conclusion:**

The findings from this study suggest that a single training session may be sufficient to learn and retain KM strike techniques relatively permanently; and the acquisition of the kick technique may lead to concomitant improvements in kick velocity and impact force.

## Introduction

The primary goal of martial arts and self-defense training is to strengthen the capacity to defend oneself against potential attacks (Cummings, 1992; Brecklin, 2008). This involves not only blocking, but also the ability to deliver strikes as quickly and powerfully as possible. Previous research has demonstrated that as experience level and training time increases, strike velocity and impact forces increase, during both punch and kick strikes (Joch, Fritche & Krause, 1981; Smith & Hamill, 1986; Sidthilaw, 1996; Falco et al., 2009; Neto et al., 2013). Practitioners typically study a given martial art, e.g., karate, jujitsu, for many years to achieve a level of proficiency. However, a premise of self-defense training is to achieve a level of competency quickly. Krav Maga (KM) self-defense experts suggest that techniques are based on simple movements that are easily learned and combined together to create entire techniques to be used for effective self-defense (Philippe, 2006). However, the idea of rapid skill acquisition of KM methods has been evaluated in only one prior study (Boe, 2015), and that study assessed only participants’ sense of confidence and level of knowledge with respect to performing self-defense techniques in close combat situations, and did not address KM performance in terms of biomechanical analysis and skilled performance. Biomechanical methods have been used to analyze the performance of martial arts experts (Fernandes et al., 2011; Gulledge & Dapena, 2008; Piorkowski, Lees & Barton, 2011; Groen, Weerdesteyn & Duysen, 2007), as well as to compare experts to amateur practitioners (Neto & Magini, 2008; Neto et al., 2013; Pucsok, Nelson & Ng, 2001; Falco et al., 2009; Bertucco & Cesari, 2008). Of the few studies that have examined the learning of martial arts, Burke et al. (2011) reported that, among 15 participants without any prior martial arts experience, an average of 29 h of training was required to learn 21 offensive and defensive techniques, drawn from Aikido, Taekwondo, Shotokan and military hand-to-hand combat. Weerdesteyn et al. (2008) taught martial arts-based lateral-fall techniques to naïve young adults during a 30-min training session and found that with this brief training session participants were able to reduce hip impact forces by as much as 17%. Gomes et al. (2016) found that the retention and transfer of a novel Judo technique was optimized under practice conditions similar to those of competition after only 8 days of 30 practice trials per day. Together, these results demonstrate the idea that a short training period can be effective for novel skill acquisition and short-term retention in martial arts. Similarly, the KM program is premised on training progressions that lead to increased striking velocity and shift of mass in the direction of the attack, ultimately resulting in maximum force production by the performer (Aviram, 2014). Only one study has examined the learning of KM in the context of a rapid learning environment (Boe, 2015). That study investigated the effects of an eight-hour training session with 43 Norwegian military officers and used pre- and post-intervention questionnaires to assess the effectiveness of the KM training on the officers’ ability to function under stress. This study demonstrated positive effects towards the efficiency of the KM system and suggests that KM is a self-defense system that quickly establishes confidence as well as increased knowledge in close combat situations for those who practice it (Boe, 2015). Furthermore, it is suggested that the increased knowledge of combat skills perceived by the officers may be attributed to how KM techniques are taught. Specifically, techniques are broken down into components or smaller steps for learning. However, that study did not examine the performance, learning or retention of techniques in terms of actual body movements, or skill development.

The initial stages of learning a novel motor skill is often marked by a rapid improvement in skill performance over a number of practice trials, best characterized as a power-law curve, even following a single training (ST) session, for a number of motor tasks (Bahrick, Fitts & Briggs, 1957; Shea & Morgan, 1979; Shea, Wulf & Whitacre, 1999; Aiken, Fairbrother & Post, 2012). For example, Aiken, Fairbrother & Post (2012) reported improved performance in basketball free throw shooting form and shooting accuracy following as little as 15 practice trials occurring on the same day, among novice adult women. Similarly, following only eight 90-s practice trials of a dynamic balance task, participants demonstrated improved balance performance on the stabilometer (Shea, Wulf & Whitacre, 1999). Such studies have demonstrated the ability of rapid skill acquisition and improved performance of a breadth of novel motor tasks in as little as a ST session among novice participants. The current study has extended these findings to include KM, which is normally taught by instructing students to perform and integrate a series of simple motor tasks to compose a complex, whole body movement.

The purpose of the current study was to examine the learning of two KM techniques: straight punch (SP), and defensive kick (DK), and the effect of multiple training (MT) sessions on skill development. We hypothesized that: (1) performance of the techniques would improve following the initial instruction and training session; and (2) performance would continue improving in the group that continued receiving instruction and training over the following several days.

## Materials and Methods

### Participants

Sixteen healthy female university students (mean ± SD; age: 23 ± 3.7 years, height: 1.65 ± 0.1 m, body mass: 62.7 ± 6.2 kg) volunteered to participate in the study. Each participant provided written informed consent prior to participation. The York University Research Ethics Review board granted approval for the study (Certificate #: 2016-367). All participants completed a screening questionnaire to assess inclusion and exclusion criteria. Inclusion criteria included: healthy young adult females between 18 and 30 years of age, and no previous martial arts/self-defense experience. Exclusion criteria included: male sex, and having experienced a musculoskeletal injury within the past 6 months that might affect the individual’s ability to perform the required movements. Participants were randomized to one of two groups: ST or MT, with eight participants assigned to each group.

### Experimental set-up and equipment

Participants wore tight-fitting black tank tops and shorts, and a headband, to facilitate motion capture. All participants were barefoot throughout testing. Body mass, height, trunk depth, knee, ankle, wrist, and elbow width were recorded. Participants completed a series of warm-up exercises guided by the experimenter to minimize the risk of injury during testing. The duration of the warm-up was 10 min, and it consisted of jumping jacks, and circular movements of the arms, wrists, neck, hips, and ankles, and deltoid and triceps stretches. A seven-camera motion capture system (Vicon, Denver, CO, USA) was used to record movement of infrared reflective markers affixed to the skin, clothing, and headband using double-sided medical-grade adhesive tape; kinematic data were sampled at 100 Hz.

**Figure 1:**
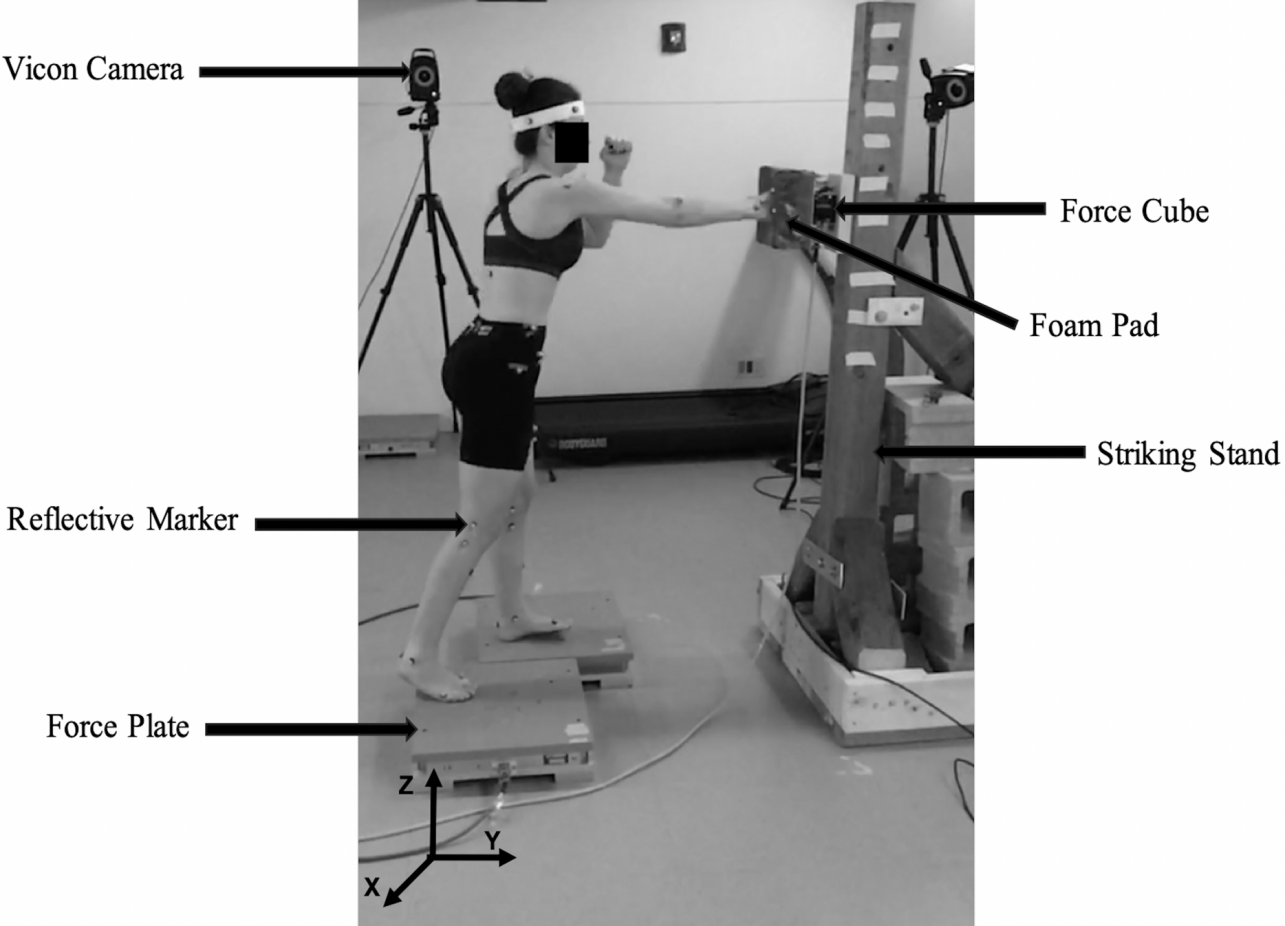
Equipment set-up. Reflective markers were affixed to each participant, which allowed the Vicon cameras to capture and record the position and change in position of each marker over time. The two force plates recorded ground reaction forces from both feet during strike movements. The force cube, padded with high-density foam, was mounted to the adjustable striking stand to record strike impact forces.

Two AMTI OR6-7-1000 force plates (AMTI, Watertown, MA, USA) and a single AMTI MC3A-1000 force cube (AMTI, Watertown, MA, USA) were used to collect ground reaction and impact force data, respectively; kinetic data were sampled at 1000 Hz. The force plates were placed in a staggered position, with the rearward plate located on the side of the preferred striking hand/foot for each participant. The force cube was fastened securely to an adjustable wooden stand (Fig. 1). A striking plate was heavily padded with foam (9 cm thick) and was attached to the force cube; the striking plate measured 21 cm by 20 cm, and participants either punched or kicked the foam-covered striking plate during each experimental trial. A small square target was placed at the center of the pad and participants were asked to aim their strikes at this target. The striking height of the target was adjusted based on the height of each participant; the punch height was positioned at shoulder height (Levine & Whitman, 2016); and the kicking height was positioned at naval/hip-level.

### Procedure

#### Baseline assessment (PreAQ; day 1)

Prior to receiving any formal instruction or training, all participants performed a baseline assessment of punch/kick strikes. Participants were asked only to perform the movements “as powerfully, quickly and accurately as possible to the center of the target.” Participants performed five trials of both punch and kick movements, which were performed as blocks of trials and counterbalanced across participants. During each testing session, participants were allowed to rest as much as desired in order to mitigate any fatigue effects.

#### Instruction and acquisition (AQ; day 1)

Following the baseline assessment, all participants received both the instruction and segmentation training regarding proper strike techniques from a certified KM instructor, co-author OB (Wightman & Linter, 1985). Segmentation training involved teaching and practicing the movements based on three checkpoints from each technique. A checkpoint is defined as a component of the entire movement that serves as an aspect of the movement on which participants focus on during the learning and skill acquisition phase. The full technique is defined by the aggregation of the checkpoints. To learn the full technique is to learn each of the checkpoints and perform them in sequence or simultaneously, as appropriate. Time allotted for learning each checkpoint during AQ was 5 min, for a total of 15 min to learn each technique. Practice included striking a hand-held target and the air.

#### Reassessment #1 (PostAQ; day 1)

Immediately following AQ, all participants again performed five trials for each technique with the same instruction set provided during PreAQ.

Training (days 2–5): The MT group repeated the instruction and training protocol each day, for four consecutive days. The expert practitioner supervised each practice session, and provided additional instruction, as needed. During this period the ST group received no further training and was instructed not to practice the techniques for the remainder of the study.

#### Reassessment #2 (RT2; day 5)

All participants again performed five trials of each technique with the same instruction set provided during PreAQ. All participants were then instructed to not practice the KM techniques.

#### Reassessment #3 (RT3; day 12)

Seven days after RT2, all participants completed the testing protocol, as previously performed in the pre-AQ, post-AQ, and RT2 sessions.

### Data processing

#### Processing raw data

All raw data were digitally filtered (Visual 3D v.5, C-motion Inc., ON, Canada) using a fourth order low pass, Butterworth filter. Based on a residual analysis approach (Winter, 2009), marker and force plate data were filtered with a cutoff frequency of 7 Hz, and force cube data were filtered with a cutoff frequency of 27 Hz. After filtering all marker position data, the following body segments were created based on the marker position data: upper arm, hand, thorax, thigh, shank and foot. From the body segments, joint angles of interest were created and defined as the orientation of one segment relative to the adjacent segment. The measures of interest were divided into: (a) performance measures, and (b) outcome measures. The performance measures included: (1) peak hand recoil velocity (m/s), (2) peak anterior/posterior (A/P) ground reaction force (GRF) (N), and (3) shoulder abduction, thigh-thorax flexion, and knee extension joint angular displacements (degrees). The outcome measures included: (1) peak impact force (N), and (2) peak hand/foot velocity (m/s). All force measures were normalized to 100% body weight.

#### Quantifying strike technique performance

**Figure 2:**
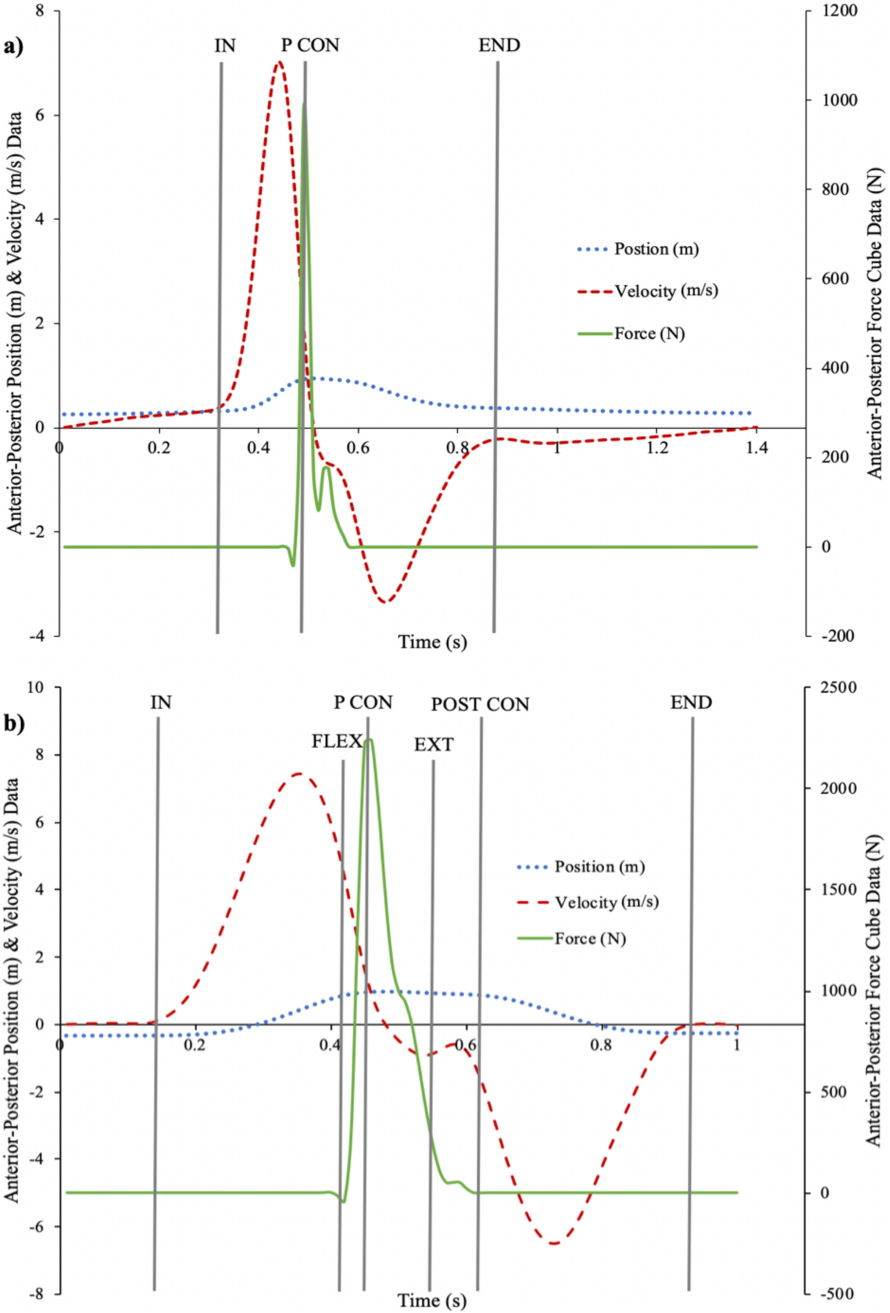
Events created within the kinematic and kinetic signals for each movement to identify and quantify performance of the individual checkpoints of both strike techniques. The plots represent the movement of an individual movement trial. (A) Straight Punch: displacement signal (blue dotted line), velocity signal (red dashed line), and impact force signal (green straight line) of striking hand in the anterior-posterior direction with events (IN = initiation of hand movement, P CON = peak force during contact with target, END = end of punch recoil). (B) Defensive Kick: displacement signal (blue dotted line), velocity signal (red dashed line), and impact force signal (green straight line) of striking foot in the anterior-posterior direction with events (IN = initiation of foot movement, P CON = peak force during contact with target, FLEX = maximum thigh-thorax flexion, EXT = maximum knee extension, POST CON = end of foot contact, END = end of kick). FLEX and EXT events are included for demonstration purposes and are based on information found in a later figure (Figs. 3D and 3E)).

**Figure 3:**
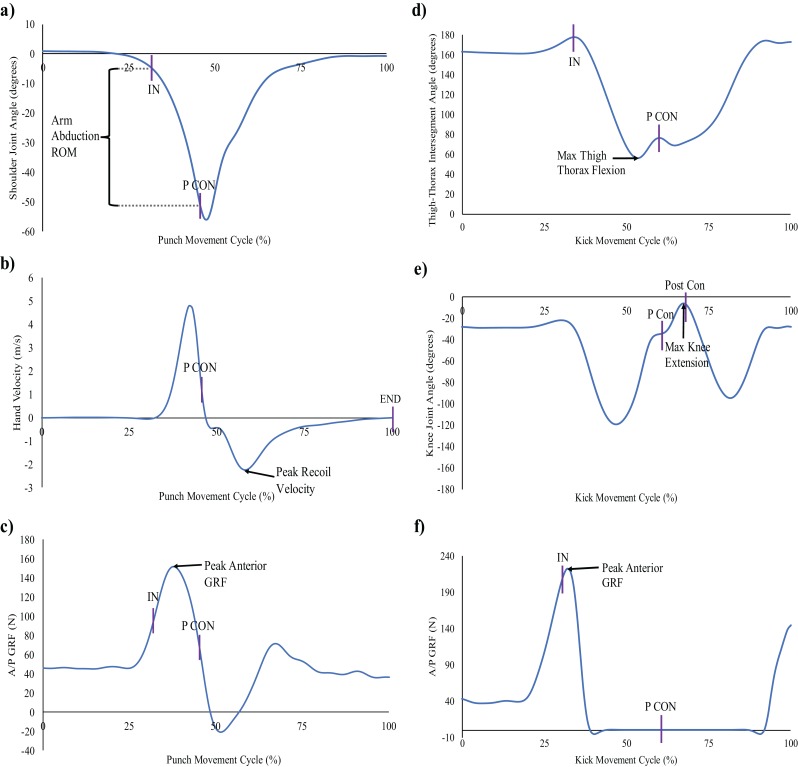
Representative plots of all punch and kick components. The plots represent the movement of an individual movement trial. Each component signal normalized to 100% of punch/kick movement between start of trial, selected at 0.5 seconds before IN (initiation of hand/foot movement), and END (end of hand/foot movement) (100%): (A) punch component 1, arm abduction ROM between the upper arm and thorax segments, calculated between IN and P CON (peak force during contact with target) events; (B) punch component 2, peak recoil velocity of the striking hand selected between P CON and END events; (C) punch component 3, peak anterior GRF, calculated between IN and P CON events; (D) kick component 1, maximum thigh-thorax flexion angle, calculated between IN and P CON events; (E) kick component 2, maximum knee extension angle, calculated between P CON and POST CON (end of foot contact) events; (F) kick component 3, peak anterior GRF, calculated between IN and P CON events. The selection of 0.5 s was used as the start of movement trial (0%) in order to capture a static posture prior to initiation of strike movement, as it became clear that the GRF production sometimes preceded both the foot and hand movements; and was therefore selected for visual purposes only.

**Figure 4:**
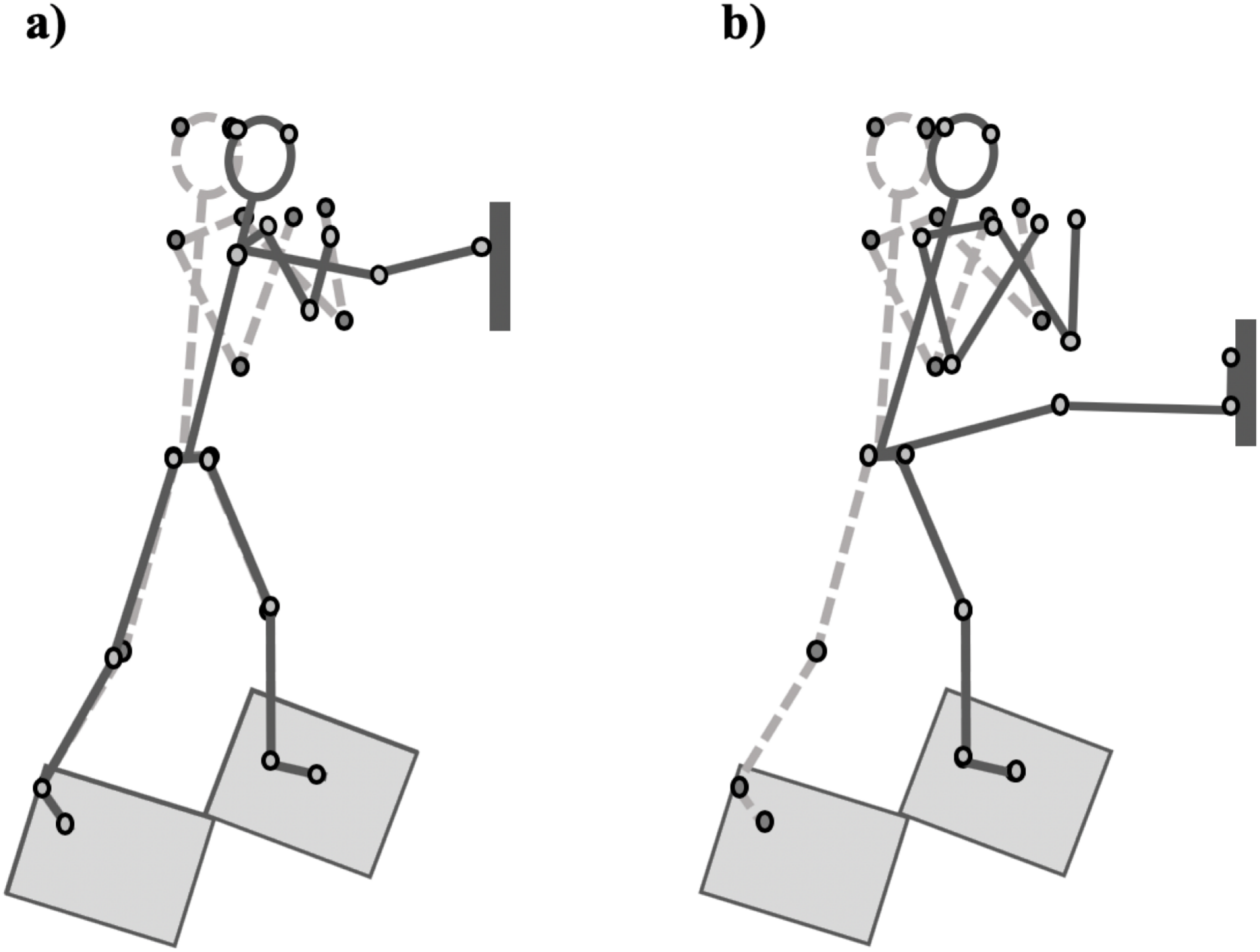
Kinematic representation of both straight punch (A) and defensive kick (B) strike techniques based on marker position data (small circles). The dashed line stick figures represent the starting position for each strike technique. The solid line stick figures represent the position at point of contact with striking surface. The light gray rectangles represent the force plates and the dark gray rectangle represents the force cube.

To quantify the performance of both techniques, specific events were created within the kinematic and kinetic signals for each movement (Fig. 2), which allowed the identification and quantification of the individual checkpoints. The measures of interest were determined on the basis of, and to be reflective of, those components (see Fig. 3). Representation of both strike techniques from initial starting position to point of contact with target is demonstrated based on marker position data in Fig. 4. The events and checkpoints, as well as the performance and outcome measures, are further described in Tables 1 and 2, respectively.

**Table 1:** Checkpoints and associated components of the Krav Maga techniques, outlined by events.

Straight punch technique			
Checkpoint/Component	Movement description	Events and performance measures for component analysis	
(1) Straight/Arm abduction	The elbow is maintained tucked in close to the side of the body, pointed towards the ground and extends as the fist travels forward towards the target during the punch. The shoulder is adducted horizontally throughout the movement until contact with target is made. The first and second metacarpal knuckles of the fist contact the target while externally rotated at about 45° from horizontal and the first metacarpal knuckle is in line with the radius of the forearm.	Event 1: IN	Initiation of hand movement indicated by first instance of positive hand velocity in the anterior-posterior direction towards the target.
		Events 2: P CON	Peak contact with target indicated by peak force on force cube in the anterior-posterior direction.
		Measure	Amount of arm abduction about the shoulder joint in degrees between events.
(2) Recoil/Peak recoil velocity	The fist is recoiled immediately and rapidly after contact with target.	Event 1: P CON	Peak contact with target indicated by peak force on force cube in the anterior-posterior direction.
		Event 2: END	End of punch recoil indicated when the hand stops moving back towards the body in the anterior-posterior direction.
		Measure	Peak velocity value of fist recoil between events.
(3) Push/Peak anterior GRF	The posterior foot pushes forcefully backward, along the A/P axis, away from the target during the initiation of the punch.	Event 1: IN	Initiation of hand movement indicated by first instance of positive hand velocity in the anterior-posterior direction towards the target.
		Event 2: P CON	Peak contact with target indicated by peak force on force cube in the anterior-posterior direction.
		Measure	Peak anterior GRF.
Defensive kick technique			
(1) Knee lift/Thigh-Thorax flexion	The dorsi flexed foot and flexed knee of the kicking leg is lifted in front (anteriorly and vertically) of the participant as high and as close to the chest as possible, where the relative joint angle, created between the thigh and thorax segments, exhibits flexion at an angle occurring at a minimum of less than 90° of flexion.	Event 1: IN	Initiation of foot movement indicated by first instance of positive foot velocity in the anterior-posterior direction towards the target.
		Event 2: P CON	Peak contact with target indicated by peak spike in force on the force cube in the anterior-posterior direction.
		Measure	Maximum thigh-thorax flexion about the thigh-thorax joint in degrees between events.
(2) Knee extension/Knee extension	The leg and hip are extended forward and horizontally adducted until the sole of the foot makes contact with the target, with an emphasis on extension of the knee, while adducted horizontally before, during and after contact with target is made.	Event 1: P CON	Peak contact with target indicated by peak spike in force on the force cube in the anterior-posterior direction.
		Event 2: POST CON	End of foot contact with target indicted by zero impact reaction force on the force cube in the anterior-posterior direction.
		Measure	Greatest extension angle about the knee joint in degrees between events.
(3) Push/Peak anterior GRF	The posterior foot pushes forcefully backward, along the A/P axis, away from the target during the initiation of the kick.	Event 1: IN	Initiation of foot movement indicated by first instance of positive foot velocity in the anterior-posterior direction towards the target.
		Event 2: POST CON	Peak contact with target indicated by peak spike in force on the force cube in the anterior-posterior direction.
		Measure	Peak anterior GRF.

**Table 2:** Outcome measures of both Krav Maga techniques, outlined by events.

Straight punch technique		Defensive kick technique	
Outcome measure 1	Peak strike velocity.	Outcome measure 1	Peak strike velocity.
Event 1: IN	Initiation of hand movement indicated by first instance of positive hand velocity in the anterior-posterior direction towards the target.	Event 1: IN	Initiation of foot movement indicated by first instance of positive foot velocity in the anterior-posterior direction towards the target.
Event 2: P CON	Peak contact with target indicated by peak force on force cube in the anterior-posterior direction.	Event 2: P CON	Peak contact with target indicated by peak spike in force on the force cube in the anterior-posterior direction.
Measure	Peak velocity value of fist between events IN and P CON.	Measure	Peak velocity value of foot between events IN and P CON.
Outcome measure 2	Peak impact force.	Outcome measure 2	Peak impact force.
Event 1: P CON	Peak contact with target indicated by peak force on force cube in the anterior-posterior direction.	Event 1: P CON	Peak contact with target indicated by peak spike in force on the force cube in the anterior-posterior direction.
Measure	Peak force value produced by fist at event P CON.	Measure	Peak force value produced by foot at event P CON.

### Statistical analysis

Statistical analyses were performed using JMP 9 (The SAS Institute, Cary, NC, USA). Mixed, repeated-measures 2 (GROUP; ST/MT) by 4 (TIMEPOINT; Baseline/RT1/RT2/RT3) analysis of variance were used to evaluate differences in the performance and outcome measures, for both KM techniques. Results were statistically significant at *p* < 0.05. Tukey’s HSD was used to compare means, and test interactions. The effect size was reported using generalized eta squared (η_G_^2^) and considered trivial (<0.02), small (0.2–0.12), moderate (0.13–0.25), and large (≥0.26) (Bakeman, 2005).

## Results

A summary of both SP technique and DK technique performance and outcome measures results is presented in Table 3.

**Table 3:** Summary of strike technique performance and outcome measures (mean ± SE) across Timepoints with groups collapsed.

	Timepoint			
	Baseline	RT1	RT2	RT3
Straight punch				
Arm abduction ROM (°)	25 ± 3	25 ± 2	27 ± 2	25 ± 3
Hand recoil velocity (m/s)	–1.7 ± 0.2	–2.2 ± 0.1	–2.3 ± 0.2	–2.2 ± 0.2
Peak anterior GRF (%BW)	14.6 ± 0.8	20.3 ± 1.4	19.3 ± 1.6	17.6 ± 1.3
Peak impact force (%BW)	74.5 ± 3.8	80.2 ± 3.6	76.9 ± 4.6	75.6 ± 3.8
Peak strike velocity (m/s)	4.3 ± 0.2	4.6 ± 0.1	4.6 ± 0.1	4.5 ± 0.1
Defensive kick				
Thigh-Thorax flexion angle (°)	–52 ± 3	–43 ± 4	–42 ± 4	–43 ± 3
Knee extension angle (°)	–28 ± 3	–21 ± 2	–19 ± 3	–20 ± 2
Peak anterior GRF (%BW)	21.0 ± 1.7	34.3 ± 2.0	33.0 ± 1.1	34.7 ± 1.7
Peak impact force (%BW)	153.5 ± 11.8	220.0 ± 16.4	225.0 ± 17.4	212.0 ± 16.6
Peak strike velocity (m/s)	4.5 ± 0.3	5.3 ± 0.2	5.0 ± 0.1	5.2 ± 0.2

**Note:**

Baseline assessment—immediately before initial training sessions; Reassessment #1 (RT1)—immediately after initial training session; Reassessment #2 (RT2)—5 days after RT1; Reassessment #3 (RT3)—7 days after RT2. Light-shade is significantly different from no-shade, and dark-shade is significantly different from both light-shade and no-shade. Significance level set to *p* < 0.05.

### Straight punch

#### Performance measures

##### Arm abduction

No significant interaction effect of Group and Timepoint (*F*(3, 42) = 1.08, *p* = 0.37, η_G_^2^ = 0.04), or main effects of Group (*F*(1, 14) = 0.10, *p* = 0.75, η_G_^2^ < 0.01) and Timepoint (*F*(3, 42) = 0.24, *p* = 0.87, η_G_^2^ < 0.01) were found for arm abduction angle ROM.

##### Recoil velocity

No significant interaction effect of Group and Timepoint (*F*(3, 42) = 0.34, *p* = 0.80, η_G_^2^ < 0.01), or main effect of Group (*F*(1, 14) = 0.64, *p* = 0.43, η_G_^2^ = 0.02) were found. A significant main effect of Timepoint (*F*(3, 42) = 5.5, *p* < 0.01, η_G_^2^ = 0.14) was found for peak recoil velocity. Post hoc analysis revealed a significant difference between the Baseline Timepoint from all other Timepoints, which were not different from each other; demonstrating a larger peak recoil velocity following AQ, which did not change for either group.

##### Anterior-posterior ground reaction force

No significant interaction effect of Group and Timepoint (*F*(3, 42) = 2.78, *p* = 0.05, η_G_^2^ = 0.06), or main effect of Group (*F*(1, 14) = 0.80, *p* = 0.39, η_G_^2^ = 0.03) was found. A significant main effect of Timepoint (*F*(3, 42) = 7.41, *p* < 0.01, η_G_^2^ = 0.15) was found for peak anterior GRF. Post hoc analysis of Timepoint revealed that Baseline was significantly different from RT1 and RT2, which were not different from each other, while RT3 was significantly different from every other timepoint; demonstrating a larger peak anterior GRF following AQ, which did not change between RT1 and RT2 testing, but declined at RT3, for both groups.

#### Outcome measures

##### Punch strike velocity

No significant interaction effect of Group and Timepoint (*F*(3, 42) = 0.60, *p* = 0.62, η_G_^2^ < 0.01), or main effects of Group (*F*(1, 14) < 0.01, *p* = 0.99, η_G_^2^ = 0.03) and Timepoint (*F*(3, 42) = 1.83, *p* = 0.15, η_G_^2^ = 0.04) were found for peak punch strike velocity.

##### Punch impact force

No significant interaction effect of Group and Timepoint (*F*(3, 42) = 2.56, *p* = 0.07, η_G_^2^ = 0.05), or main effects of Group (*F*(1, 14) = 1.38, *p* = 0.26, η_G_^2^ = 0.05) and Timepoint (*F*(3, 42) = 1.12, *p* = 0.35, η_G_^2^ = 0.02) were found for peak punch impact force.

### Defensive kick

#### Performance measures

##### Thigh-thorax flexion angle

No significant interaction effect of Group and Timepoint (*F*(3, 42) = 0.51, *p* = 0.07, η_G_^2^ < 0.01), or main effect of Group (*F*(1, 14) = 0.62, *p* = 0.44, η_G_^2^ = 0.03) was found. A significant main effect of Timepoint (*F*(3, 42) = 4.43, *p* < 0.01, η_G_^2^ = 0.08), was found for maximum thigh-thorax flexion angle. Post hoc analysis of Timepoint revealed that Baseline was significantly different from all other Timepoints, which were not different from each other; demonstrating a larger thigh-thorax flexion angle following AQ, which did not change for either group.

##### Knee extension

No significant interaction effect of Group and Timepoint (*F*(3, 42) = 1.06, *p* = 0.38, η_G_^2^ < 0.01), or main effect of Group (*F*(1, 14) = 1.42, *p* = 0.25, η_G_^2^ = 0.04) was found. A significant main effect of Timepoint (*F*(3, 42) = 4.48, *p* < 0.01, η_G_^2^ = 0.13) was found for maximum knee extension angle. Post hoc analysis of Timepoint revealed that Baseline was significantly different from all other Timepoints, which were not different from each other; demonstrating a larger maximum knee extension angle following AQ, which did not change for either group.

##### Anterior-posterior ground reaction force

No significant interaction effect of Group and Timepoint (*F*(3, 42) = 1.27, *p* = 0.30, η_G_^2^ = 0.02), or main effect of Group (*F*(1, 14) = 0.07, *p* = 0.79, η_G_^2^ < 0.01) was found. A significant main effect of Timepoint (*F*(3, 42) = 22.8, *p* < 0.01, η_G_^2^ = 0.45) was found for peak anterior GRF. Post hoc analysis revealed that Baseline was significantly different from all other Timepoints, which were not different from each other; demonstrating a larger peak anterior GRF following AQ, which did not change for either group.

#### Outcome measures

##### Kick strike velocity

No significant interaction effect of Group and Timepoint (*F*(3, 42) = 2.44, *p* = 0.08, η_G_^2^ = 0.06), or main effect of Group (*F*(1, 14) = 0.16, *p* = 0.69, η_G_^2^ < 0.01) was found. A significant main effect of Timepoint (*F*(3, 42) = 6.98, *p* < 0.01, η_G_^2^ = 0.15) was found for peak kick strike velocity. Post hoc analysis of Timepoint revealed that Baseline was significantly different from all other Timepoints, which were not different from each other; demonstrating a larger peak kick strike velocity following AQ, which did not change for either group.

##### Kick impact force

No significant interaction effect of Group and Timepoint (*F*(3, 42) = 0.87, *p* = 0.46, η_G_^2^ < 0.01), or main effect of Group (*F*(1, 14) = 0.93, *p* = 0.35, η_G_^2^ = 0.04) was found. A significant main effect of Timepoint (*F*(3, 42) = 18.2, *p* < 0.01, η_G_^2^ = 0.19) was found for peak kick impact force. Post hoc analysis of Timepoint revealed that Baseline was significantly different from all other Timepoints, which were not different from each other; demonstrating a larger peak kick impact force following AQ, which did not change for either group.

## Discussion

The current study investigated the learning of two KM techniques: SP and DK, and the effect of MT sessions on skill acquisition. Results from each performance measure demonstrated that all three checkpoints for the DK technique, and two of the three checkpoints of the SP technique, improved following AQ, which partially confirmed the first hypothesis that performance of the two KM techniques would improve with a single session of instruction. Furthermore, the outcome measures, peak strike velocity and impact force, similarly improved with AQ but only for the DK technique; no changes were observed in either peak impact force or peak strike velocity for the SP technique. These findings suggest that both techniques can be learned in as little as 15 min of training, but that with limited instruction and training, increases in punch velocity and impact force may not be observed. Furthermore, beyond AQ, no further improvements were observed for either group, which is notable because one of the groups, the MT group, received four additional training sessions.

Results from the MT group demonstrated that performance did not continue to improve; therefore, the second hypothesis was rejected. Results from the ST group demonstrated that performance of both techniques did not degrade between PostAQ and RT2. The absence of decline in the performance found for the ST group was surprising as participants in this group were instructed not to practice for the remainder of the study. However, the absence of improvement in the performance of the participants in the MT group, all of whom received MT sessions between PostAQ and RT2, was also unanticipated. The performance of the checkpoints also did not differ between RT2 and RT3, except for peak anterior GRF, which demonstrated a significant decrease in performance. These findings suggest that the skill level obtained in the performance of the SP and the DK during AQ was generally maintained for a period of up to 12 days, even for the group that received no additional training after AQ.

### Performance measures

#### Multiple training group

The absence of continued improvement in performance and outcome measures for the MT group might be explained by a number of factors, rooted in theories of motor learning. First, it is possible that the volumes of practice provided (i.e., 15 min per strike technique over four consecutive days), was insufficient to improve skill level. These findings may be in accordance with the power law of practice, which states that learning occurs at a rapid rate at the onset of practice but that the rate of learning decreases as practice increases over time (Chapman, 1919; Newell & Rosenbloom, 1981). In this study, the skill level of participants improved rapidly after AQ, as participants learned the basic movements and expectations of performance. It is possible that performance levels did not change with the additional four training sessions because a learning plateau may have been reached and that much more practice may be necessary for further improvements.

Research has demonstrated that high contextual interference, stimulated by randomly alternating between tasks for each trial during training, or variable practice, results in enhanced motor skill retention, as opposed to a blocked schedule in which all trials from one task are practiced before moving on to the next task (Goode & Magill, 1986; Pollatou et al., 1997). The current study utilized a blocked training approach, in which the SP instruction and training was completed before participants moved on to the DK instruction and training. Alternating between punch and kick trials during practice sessions might have stimulated further improvements in the performance of both techniques in the MT group.

Previous research has demonstrated that dyad training, or training participants in a pair, is associated with greater learning outcomes, compared to training individually, as this mode of training permits the learner to experience and benefit from additional processing through observational learning during periods when the partner practices. This combination of observational learning and physical practice may enable the observer to extract additional information about the task, such as appropriate coordination patterns, which may be missed during physical practice alone (Shebilske et al., 1992). Furthermore, increased motivation has also been found during dyad training, due to the stimulation of competition between participants during training (Wulf, Shea & Lewthwaite, 2010). Including a dyad training approach in the training protocol of the current study might have resulted in further improvement in the MT group.

Self-controlled training provides the participant with control over their own learning, during the practice session, leading to a more active role and increased motivation; as well as a tailored practice experience to meet their individual needs (Chiviacowsky et al., 2008; Post, Fairbrother & Barros, 2011). The highly structured and instructor-controlled KM training protocol may have been beneficial when first learning the skills, however, as participants in the MT group continued training throughout the study, it is possible that they felt “forced” into a given practice schedule and volume of practice prescribed by the highly contrived experiment-based context for training. In summary, we speculate that the contrived training protocol of the study itself might have limited any improvement in performance beyond AQ when the participants transitioned from being utterly naïve to the practice of KM.

The lack of performance improvements at RT2 among participants in the MT group with continued training between RT1 and RT2 might be explained by the low volume of practice during this period, but also the absence of the incorporation of motor learning theories such as dyad training and variable practice, described above. Future research should investigate the use of different training paradigms and the combination of training paradigms, to encourage continued performance improvement with ongoing training among novice performers.

#### Single training group

The findings for the ST group suggested that skill level of the DK technique did not degrade for a period of up to 12 days, with no additional training. Proponents and practitioners of KM emphasize that techniques might be easier to learn, compared with other combat and martial arts techniques, because the techniques are composed of simple movements. The relative simplicity of learning and performing the movements allows for a basic level of skill to be obtained quickly and retained relatively permanently. Weerdesteyn et al. (2008) demonstrated that teaching martial arts falls techniques to naïve participants in a brief 30-min instruction session had a significant effect in reducing hip impact forces with the ground. Additionally, when inexperienced fallers were asked to perform the martial arts fall technique a few weeks after training, they demonstrated the correct performance as originally taught. This finding is in line with the results obtained in the ST group, suggesting that the relatively simple movements learned during a brief KM training session could be learned quickly and retained relatively permanently.

The lack of skill degradation in the ST group is in contrast with the results of Burke et al. (2011) who reported that maintaining proficiency in even simple martial arts techniques, such as the ready stance, would require continual practice. Although Burke et al. (2011) examined the learning curves of offensive and defensive techniques, drawn from four different martial arts systems, the techniques selected did not include KM techniques. The current results might suggest that KM techniques are more easily learned and retained, compared to techniques drawn from other martial arts systems, perhaps underscoring the assertion by KM proponents that the learning of KM is facilitated by the simple movements of the activity. The implications of the findings for the ST group are that a brief initial training session may be sufficient for untrained performers to learn and retain KM strike techniques relatively permanently.

### Outcome measures

The improvements in peak impact force and peak strike velocity, for the DK technique, are in line with the primary objective of learning self-defense, which is to increase the capacity to strike quickly and with force (Cummings, 1992; Brecklin, 2008). Previous research has demonstrated that learning and practicing martial arts techniques results in an increase in striking limb velocity, ultimately resulting in increased impact forces (Neto & Magini, 2008; Roberts et al., 2013). In the current study, the acquisition of the DK technique, as evidenced by an improvement in the performance measures, demonstrated an improvement in peak kick velocity and peak impact force. These findings suggest the following progression: (i) DK technique was learned, (ii) the learned technique and successful performance of the checkpoints, resulted in increased peak strike velocity, and (iii) increased peak strike velocity may have resulted in increased peak impact force in the kick technique. However, even though improvement in performance of two of the three checkpoints in the SP technique was demonstrated, neither impact force or strike velocity were observed to improve. A clear limitation of this study is the immovability of the striking target. Although the target was heavily padded with foam, the target remained static during impact trials. Therefore, it is possible that the participants self-regulated the velocity and impact force of the punch technique for fear of injury. The hand and wrist are relatively weak structures, compared with the robustness of the foot, ankle, and lower leg. Perhaps increasing the duration of practice, in terms of the length of each session, and the number of days of practice, would permit a level of comfort with the punching technique that would reduce such self-regulation. Additionally, perhaps modifying the design of the striking target such that the target may be placed on a mechanical shock absorber when the impact force is applied, reducing discomfort and risk of injury. Future research should investigate the possibility of a relationship between fear of injury and performance of punching techniques.

## Conclusions

The current study examined the motor skill acquisition and retention of two KM self-defense strike techniques: SP and DK under two training conditions, in untrained females. The results demonstrated no significant difference in learning or performance of the techniques between two groups that received either a single instruction and training session, or multiple sessions, for both techniques. Further, the skill level obtained following AQ, can be retained for up to 12 days. It is speculated that the dynamics of learning the SP technique are different than those of the DK technique, perhaps related to fear of injury, suggesting that additional training may be required for the SP technique. It is also speculated that the additional training sessions did not provide enough learning to achieve further improvements in the performance of either technique after AQ. Instructors and practitioners might consider that additional training, in terms of increased session duration and number of sessions, could be required to stimulate an improvement in performance. As well, restructuring of the training protocol following AQ may serve to produce further improvement in technique performance. Suggestions include dyad training, self-controlled training, as well as increasing contextual interference. This study has, however, demonstrated that the SP and DK techniques can be learned in as little as a single 15-min instruction session, with commensurate improvements in measures of performance of the checkpoints, and the outcome measures of strike velocity and impact force, understanding that there seem to be differences in acquiring the SP, compared with the DK, that remain to be explored.

## Supplemental Information

10.7717/peerj.8525/supp-1 Supplemental Information 1 Performance and outcome measures data of each movement trial for both the straight punch and defensive kick techniques.

10.7717/peerj.8525/supp-2 Supplemental Information 2 Checkpoints and associated components of the Krav Maga techniques, outlined by events.

10.7717/peerj.8525/supp-3 Supplemental Information 3 Outcome measures of both Krav Maga techniques, outlined by events.

10.7717/peerj.8525/supp-4 Supplemental Information 4 Summary of strike technique performance and outcome measures (mean ± SE) across Timepoints with groups collapsed. Baseline assessment - immediately before initial training sessions; Reassessment #1 (RT1) - immediately after initial training session; Reassessment #2 (RT2) - 5 days after RT1; Reassessment #3 (RT3) - 7 days after RT2. Light-shade is significantly different from no-shade, and dark-shade is significantly different from both light-shade and no-shade. Significance level set to *p* < 0.05.

## Additional Information and Declarations

### Data Availability

The following information was supplied regarding data availability: The raw data are available in the Supplemental Files.
